# Single-Cell Transcriptomic Analysis of Peripheral Blood Reveals a Novel B-Cell Subset in Renal Allograft Recipients With Accommodation

**DOI:** 10.3389/fphar.2021.706580

**Published:** 2021-09-30

**Authors:** Quan Zhuang, Hao Li, Bo Peng, Yang Liu, Ying Zhang, Haozheng Cai, Shu Liu, Yingzi Ming

**Affiliations:** ^1^ Transplantation Center, The Third Xiangya Hospital, Central South University, Changsha, China; ^2^ Research Center of National Health Ministry on Transplantation Medicine, Changsha, China

**Keywords:** kidney transplantation, single-cell RNA sequencing, B cell, immune accommodation, flow cytometry

## Abstract

**Background:** Kidney transplantation (KTx) is a preeminent treatment for end-stage renal disease (ESRD). After the application of immunosuppressants (IS), renal allograft recipients could reach a state called accommodation which means they are neither rejected nor infected. This study aimed to describe the details of this immune accommodation and reveal a novel mechanism of IS on immune cell subpopulations.

**Methods:** We analyzed multiple cell subgroups and their gene expression of peripheral T, B, myeloid, and NK cells from renal allograft recipients with accommodation and healthy control (HC) by single-cell transcriptomics sequencing (scRNA-seq) and flow cytometry.

**Results:** A total of 8,272 cells were isolated and sequenced from three individuals, including 2,758 cells from HC, 2,550 cells from ESRD patient, and 2,964 cells from KTx patient, as well as 396 immune response–related genes were detected during sequencing. 5 T-cell, 4 NK-cell, 5 myeloid, and 4 B-cell clusters were defined. Among them, a B-cell subset (CD19^+^IGLC3^low^IGKC^high^TCL1A^-^CD127^+^) of renal transplant recipients with accommodation was significantly lower than that of HC and verified by flow cytometry, and this B-cell subset showed an activated potential because of its high expression of CD127. Furthermore, we found that IL32 might be the key cytokine to induce the differentiation of this B-cell cluster.

**Conclusion:** We found a novel B-cell subset (CD19^+^IGLC3^low^IGKC^high^TCL1A^-^CD127^+^) which was inhibited and decreased in renal allograft recipients with accommodation. This study might reveal the effect of commonly used IS in clinical practice on B-cell subsets and related mechanism.

## Introduction

Kidney transplantation (KTx) is a preeminent treatment for end-stage renal disease (ESRD) (Parajuli et al., 2018) that effectively improves the quality of life of patients undergoing dialysis. Although the application of immunosuppressants (IS) reduces the risk of rejection in transplant patients and significantly improves the survival rates of transplant grafts and recipients, excessive IS administration creates higher risks of infection and tumor occurrence (Fishman, 2013). Finding the best balance between rejection and infection is an important issue faced by every transplant doctor. In clinical practice, most transplant recipients can reach a state called accommodation (Garcia de Mattos Barbosa et al., 2018), a state in which neither rejection nor infection occurs post transplantation. However, accommodation is still different from the immune state of healthy people and is a special type of immune homeostasis under immunosuppressive conditions.

Currently, IS commonly used in the clinic (i.e., FK506, mycophenolate mofetil (MMF), and steroids) target the activation and proliferation of T cells (Romano et al., 2019). However, it is not clear whether these IS could affect other immune cells, such as B cells, NK cells, and myeloid cells. In addition, there is little research on the effects of IS on various T-cell subpopulations. Our previous study suggested that compared with healthy controls (HC), renal transplant recipients with accommodation had reduced proportions of γδ and Vδ2 subsets, as well as CD27^+^CD28^+^ subsets in both the CD4^+^ and CD8^+^ T-cell compartments, but the programmed cell death protein (PD) 1^+^ CD4^+^ and CD8^+^ T-cell subsets were increased. Additionally, an increased percentage of CD4^+^ effector memory T cells and a decreased fraction of CD8^+^ central memory T cells were found in the renal allograft recipients with accommodation (Zhuang et al., 2019). We also reported that multiple subpopulations of B cells were altered in renal allograft recipients with accommodation, including reduced levels of regulatory B cells (Bregs) (CD38^high^CD27^+^CD24^+^), transitional B cells (IgM^+^CD38^high^CD24^high^), and marginal zone (MZ) B cells but increased levels of IgD^-^CD27^+^ and CD38^low^CD21^-^ B cells (Zhuang et al., 2020).

Although allograft biopsy is the gold standard for diagnosis of transplant rejection and immune-related problems, it is difficult to scale up in accommodation patients because of its invasive nature (Snijders et al., 2020). At present, peripheral blood is still the most convenient and quickest tissue for sampling in humans, owing to its highly informative populations of immune cells and the relative harmlessness of collection. The immune distribution in the peripheral blood in renal transplant recipients with accommodation definitely varies from that in healthy people because of the application of IS, so a better understanding of the peripheral immune cell profile is needed. With the rapid development of single-cell transcriptomic sequencing (scRNA-seq) in recent years, an increasing number of new immune cell subsets and their new functions of existing immune cells have been revealed (Dulken et al., 2019; Pont et al., 2019; Xiong et al., 2019). This technology compensates for the shortcomings of bulk RNA sequencing and microarrays and can detect and analyze the comprehensive transcriptome of each immune cell subset in different diseases. scRNA-seq can even identify rare cell subpopulations that were overlooked previously (Dulken et al., 2019). scRNA-seq can analyze more than 10,000 single-cell transcriptomes once, distinguish two similar subgroups within one kind of immune cell and trace the trajectory of cell evolution (Pont et al., 2019).

In this study, we analyzed multiple cell subgroups within peripheral T, B, myeloid, and NK-cell populations from renal transplant recipients with accommodation and healthy people by scRNA-seq and flow cytometry and found that the level of a novel B-cell subset (CD19^+^IGLC3^low^IGKC^high^TCL1A^-^CD127^+^) in the renal transplant recipients with accommodation was significantly lower than that in the healthy people, and this B-cell subset showed an activated potential because of its high expression of CD127. This study might reveal the effect of IS commonly used in clinical practice on B-cell subsets and the related mechanism.

## Materials and Methods

### Human Specimens

Peripheral blood mononuclear cells (PBMCs) from two patients (one KTx patient and one ESRD patient) and one healthy volunteer were sent for scRNA-seq. The patient with KTx was in accommodation at 1 year post transplantation. The patient with ESRD was still undergoing regular hemodialysis. PBMCs from 12 KTx patients and 20 HC with backgrounds similar to those of the patients evaluated by scRNA-seq were recruited for the analysis by flow cytometry. In brief, peripheral blood was collected into EDTA-anticoagulant tubes. After red blood cell lysis, a lymphocyte separation medium (TBD and LTS1077), blood, and 1X phosphate-buffered saline (HyClone and SH30256.01) were slowly added into centrifuge tubes separately at a ratio of 2:1:1. After centrifugation at 450 g for 20 minutes (min), PBMCs were aspirated from the interface in the centrifuge tubes. The study was reviewed and approved by the Institutional Review Board (Ethics Committee) of the Third Xiangya Hospital, Central South University (No. 2018-S347). Supplementary Tables S1, S2 contain detailed information of the patients and healthy volunteers.

### scRNA-Seq Analysis

A BD Rhapsody Single-Cell Analysis System (BD Biosciences) was used for the scRNA-seq analysis. In brief, PBMCs were processed into a single-cell suspension and loaded into a BD Rhapsody cartridge with >200,000 microwells. Then, a bead library was loaded into the microwell cartridge to saturation, and each cell was paired with a microbead. Next, the bead-cell complexes were hybridized with mRNA molecules to capture the barcoded oligos on the beads after lysing the cells in the microwell cartridge. The beads were collected into a single tube to generate a multiplex PCR-based library customized by the BD Rhapsody Immune Response Targeted Panel for Human (BD Biosciences). Fastq files were processed by an Illumina HiSeq 3000 platform and then processed into the expression matrix Fastq by the BD Rhapsody Analysis Pipeline. BD DataView software (BD Biosciences) and the R package Seurat (V 4.01) were used to analyze the expression matrix. The Kyoto Encyclopedia of Genes and Genomes (KEGG) analysis was performed using the R package “enrichplot” (Yu et al., 2012). The raw expression data from these experiments are available at the NCBI Gene Expression Omnibus database, with the following identifier: GSE175429 (https://www.ncbi.nlm.nih.gov/geo/query/acc.cgi?acc=GSE175429).

### Leukocyte Staining and Flow Cytometric Analysis

The following antibodies specific for surface antigens were used: anti-human (Hu) lambda light chain-APC (eBioscience, Catalog Number 17-9990-42), anti-Hu kappa light chain-super bright 600 (eBioscience, Catalog Number 63-9970-42), anti-Hu CD3-Alexa Fluor 700 (eBioscience, Catalog Number: 56-0038-42), Anti-Hu CD4-eFluor 450 (eBioscience, Catalog Number: 48-0049-42), anti-Hu CD14-APC/cyanine7 (BioLegend, Catalog Number: 325620), anti-Hu CD19-PerCP-Cyanine5.5 (Invitrogen, Catalog Number:45-0199-42), anti-Hu CD127-PerCP-Cyanine5.5 (Invitrogen, Catalog Number:45-1278-42), Anti-LEF1-Alexa Fluor® 647 (abcam, Catalog Number: ab246715), anti-Hu TCL1-PE-Cyanine7 (Invitrogen, Catalog Number: 2132361), anti-Hu CCL4-Alexa Fluor 488 (eBioscience, Catalog Number: 25-6699-42), BD anti-CD19-PE (BD, Catalog Number: 349209), and BD Multitest 6-Color TBNK (BD, Catalog Number: 662967). For intracellular and nuclear antigens, the FOXP3/Transcription Factor Staining Buffer Set (eBioscience, Catalog Number: 00-5523-00) and a stain for nucleation treatment were used. BD Trucount Tubes (BD, Catalog Number: 340334) were used to determine the absolute cell count. FlowJo V 10.62 was used to analyze flow cytometric data. Based on the FSC-SSC plot, lymphocytes were well separated. CD3 and CD19 were used to distinguish T cells and B cells from total lymphocytes. Then, IGLC3^low^IGKC^high^ cells were gated, and the TCL1-FMO tube was used as a negative control group to identify TCL1^-^IGLC3^low^IGKC^high^ B cells.

### Statistical Analysis

The mean ± standard deviation (SD) was used to describe the analyzed data. Differences in CD19^+^IGLC3^low^IGKC^high^TCL1A^-^CD127^+^ B-cell subset percentages between groups were compared using the Mann–Whitney U-test because not all of the parameters were normally distributed. GraphPad Prism 7.0 (GraphPad Software Inc., La Jolla, CA, United States) was used to perform statistical analyses. Values of *p* < 0.05 were considered statistically significant.

## Results

### scRNA-Seq Revealed the Landscape of Peripheral Immune Cells and Novel Gene Expression Patterns

**FIGURE 1:**
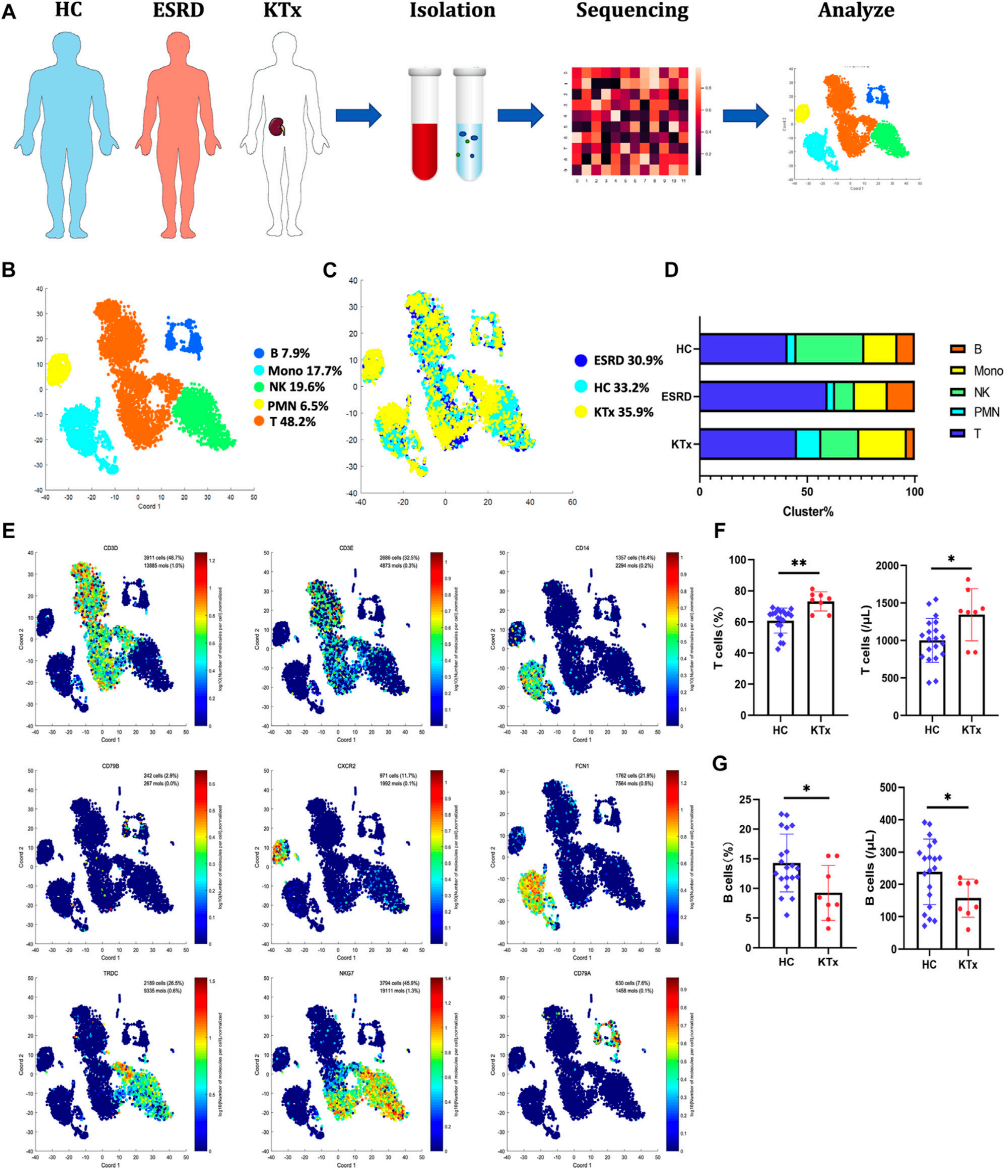
Single-cell transcriptional profiling of PBMCs from HC, ESRD, and KTx. **(A)** Schematic of experimental workflow for defining and comparing PBMCs between three donors. **(B,C)** tSNE of single-cell profile annotated by a sample type and associated cell type. **(D)** Different expressions of marker genes in different clusters above. **(E)** Fraction of cells for 5 cell types in HC, ESRD, and KTx individuals. **(F)** Proportion and absolute counts of CD3^+^ T cells in HC and KTx groups. **(G)** Ratio and absolute counts of CD19^+^ B cells in HC and KTx groups. *p < 0.05 **p < 0.005.

A total of 8,272 cells were isolated and sequenced from three individuals including 2,758 cells from the HC, 2,550 cells from the ESRD patient and the 2,964 cells from KTx patient, and 396 immune response-related genes were detected during sequencing (Figure 1A). The Seurat package was used to identify 5 distinct cell clusters across all three individuals (Figures 1B,C): a T-cell cluster (defined by CD3D and CD3E, 48.2%), a B-cell cluster (defined by CD79A and CD79B, 7.9%), a polymorphonuclear cell (PMN) cluster (defined by CXCR2, 6.5%), a monocyte cluster (defined by FCN and CD14, 17.7%), and an NK-cell cluster (defined by NKG7 and TRDC, 19.6%) (Figure 1D; Supplementary Figure S1A). Other differentially expressed genes in each patient are displayed in a heat map (Supplementary Figure S1B).

T cells are often regarded as the target cells of immunosuppressive agents (Lim et al., 2017), but in our data, B cells, not T cells, were obviously suppressed in the KTx group compared with the HC group (Figure 1G). The proportions of NK-cell and myeloid subsets were not significantly different. To verify this, we used flow cytometry to evaluate the percentages and absolute numbers of T cells (defined by CD3^+^), B cells (defined by CD19^+^), and NK cells (defined as CD16^+^CD56^+^) in the peripheral blood of healthy people and kidney transplant recipients with accommodation at 1 year after transplantation. Compared to those in the HC group, the percentage (*p = 0.019*) and absolute number (*p = 0.042*) of B cells in the transplant group were significantly decreased, while those of T cells were increased (percentage: *p = 0.0006* and absolute number: *p = 0.0136*) (Figures 1F,G). NK-cell data were consistent with the sequencing data and did not show significant differences between the KTx and HC groups (ratio: *p = 0.87* and absolute number: *p = 0.56*) (Supplementary Figure S2).

### PBMC scRNA-Seq Identified Five T-Cell Subsets

**FIGURE 2:**
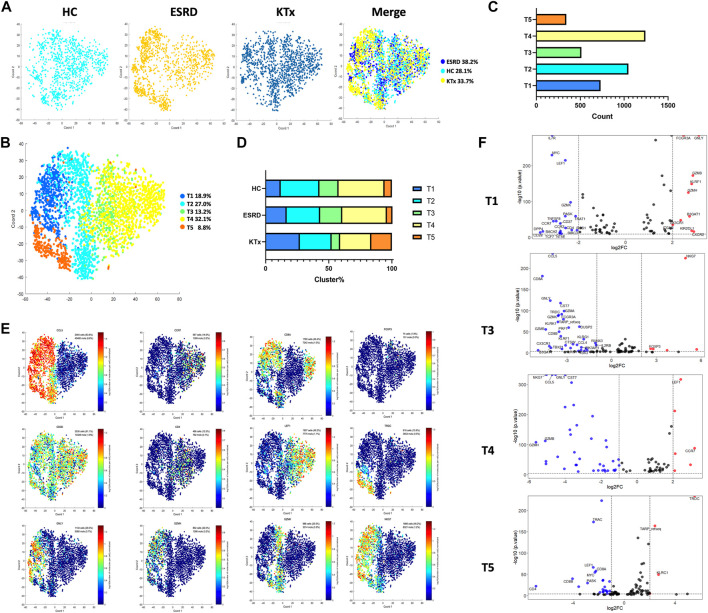
T-cell clusters in PBMCs from HC, ESRD, and KTx. **(A,B)** tSNE of single-cell profile with each cell color coded for T-cell subsets. **(C)** Cell count of 5 T-cell subsets. **(D)** Distribution of 5 T-cell subsets. **(E)** Expression of highly expressed genes in each T-cell cluster. **(F)** Gene fingerprint expression volcano map of T1, T3, T4, and T5 clusters.

The T-cell cluster distribution among the three groups is shown (Figure 2A). Two CD8^+^, two CD4^+^, and one CD8^-^CD4^-^ T-cell subpopulations were identified as follows: the T1 cluster (CD8A^+^GNLY^+^GZMH^+^GZMB^+^), T2 cluster (CD8A^+^GZMK^+^NKG7^+^), T3 cluster (CD4^+^LEF1^low^NKG7^−^), T4 cluster (CD4^+^LEF1^high^CCR7^+^NKG7^-^), and T5 cluster (CD4^-^CD8^-^TRDC^+^NKG7^+^) (Figures 2B,C). The specific gene markers and distribution are shown in Figure 2E, Supplementary Figures S3A,B. Compared with those in the HC group, the percentages of the T3 and T4 subsets in the KTx group were decreased significantly, while the proportion of the T1 and T5 subset in the KTx group was obviously increased (Figure 2D). The results of KEGG and GO enrichment analyses of highly expressed genes in the T3 subset showed a positive regulation of lymphocyte activation (Supplementary Figure S3C); however, the FOXP3 gene was upregulated in the T3 subset compared to the other T-cell subsets (Figure 2E). According to the gene expression pattern of the T5 subset (Figure 2F; Supplementary Figures S3A,B), we considered the T5 subset to be γδ T cells, and the results of KEGG and GO enrichment analyses of highly expressed genes in the T5 subset showed that this subset was related to the adaptive immune system (Supplementary Figure S3D).

### PBMC scRNA-Seq Identified Four NK-Cell Subsets

**FIGURE 3:**
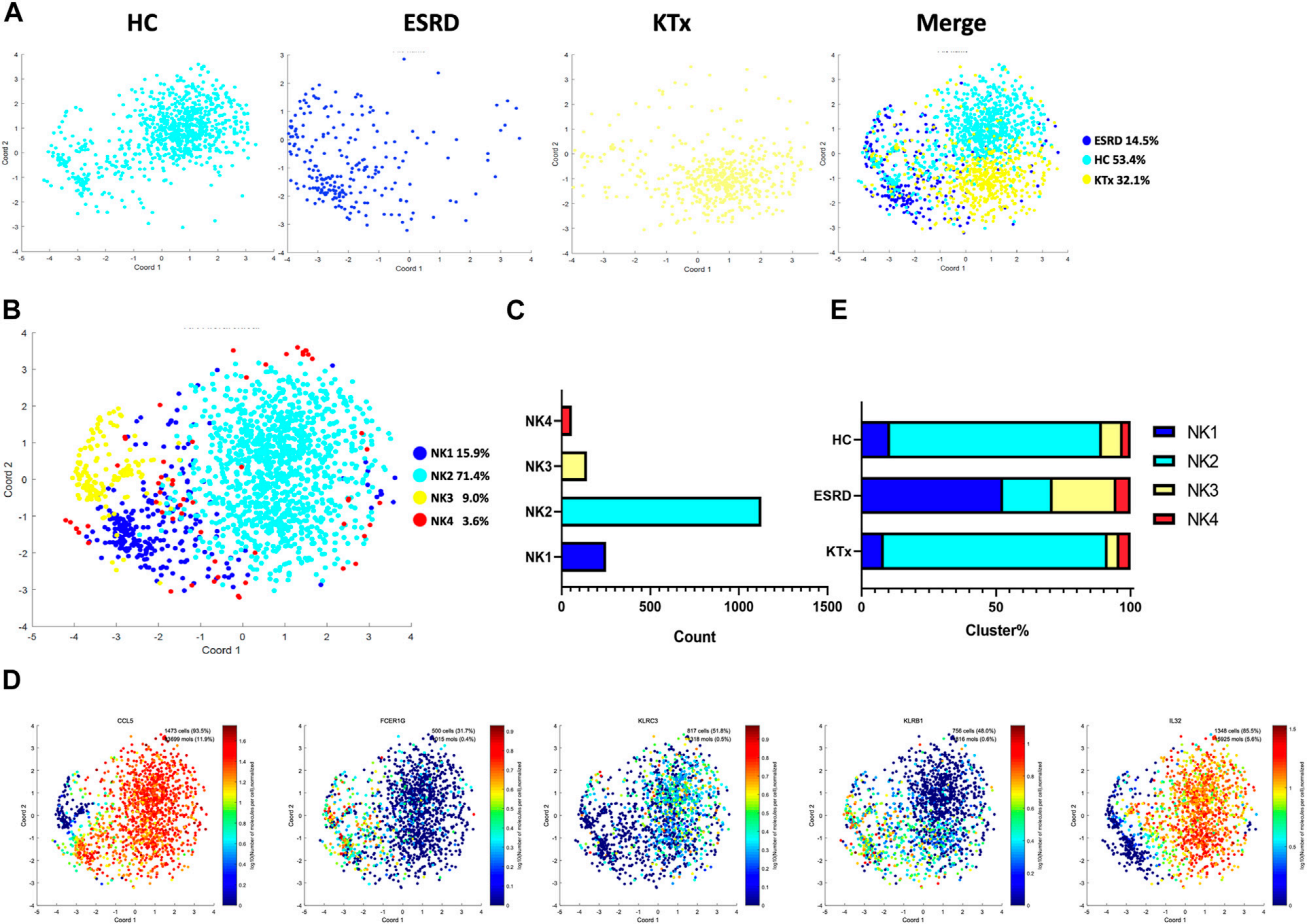
NK-cell clusters in PBMCs from HC, ESRD, and KTx. **(A,B)** tSNE of single-cell profile with each cell color coded for NK-cell subsets. **(C)** Cell count of 4 NK-cell subsets. **(D)** Expression of highly expressed genes in each NK-cell cluster. **(E)** Proportion of 4 NK-cell subsets.

Four NK-cell subpopulations defined by scRNA-seq (Figures 3A,B) were as follows: the NK1 cluster (FCER1G^+^CCL5^+^), NK2 cluster (IL32^+^KLRC3^+^), NK3 cluster (FCER1G^+^CCL5^-^), and NK4 cluster (IL32^+^KLRB^+^) (Figure 3C). The specific gene markers and distribution are shown in Figure 3D, Supplementary Figures S4A,B. Figure 3E shows that there was no significant difference in the cell fraction between the KTx and HC groups. The flow cytometry analysis also proved that NK cells (CD16^+^CD56^+^) were not significantly different between these two groups (Supplementary Figure S2). In contrast, the ESRD group showed high proportions of the NK1 and NK3 subgroups but a low proportion of the NK2 subgroup compared to the other two groups (Figure 3E).

### PBMC scRNA-Seq Identified Five Myeloid Cell Subsets

**FIGURE 4:**
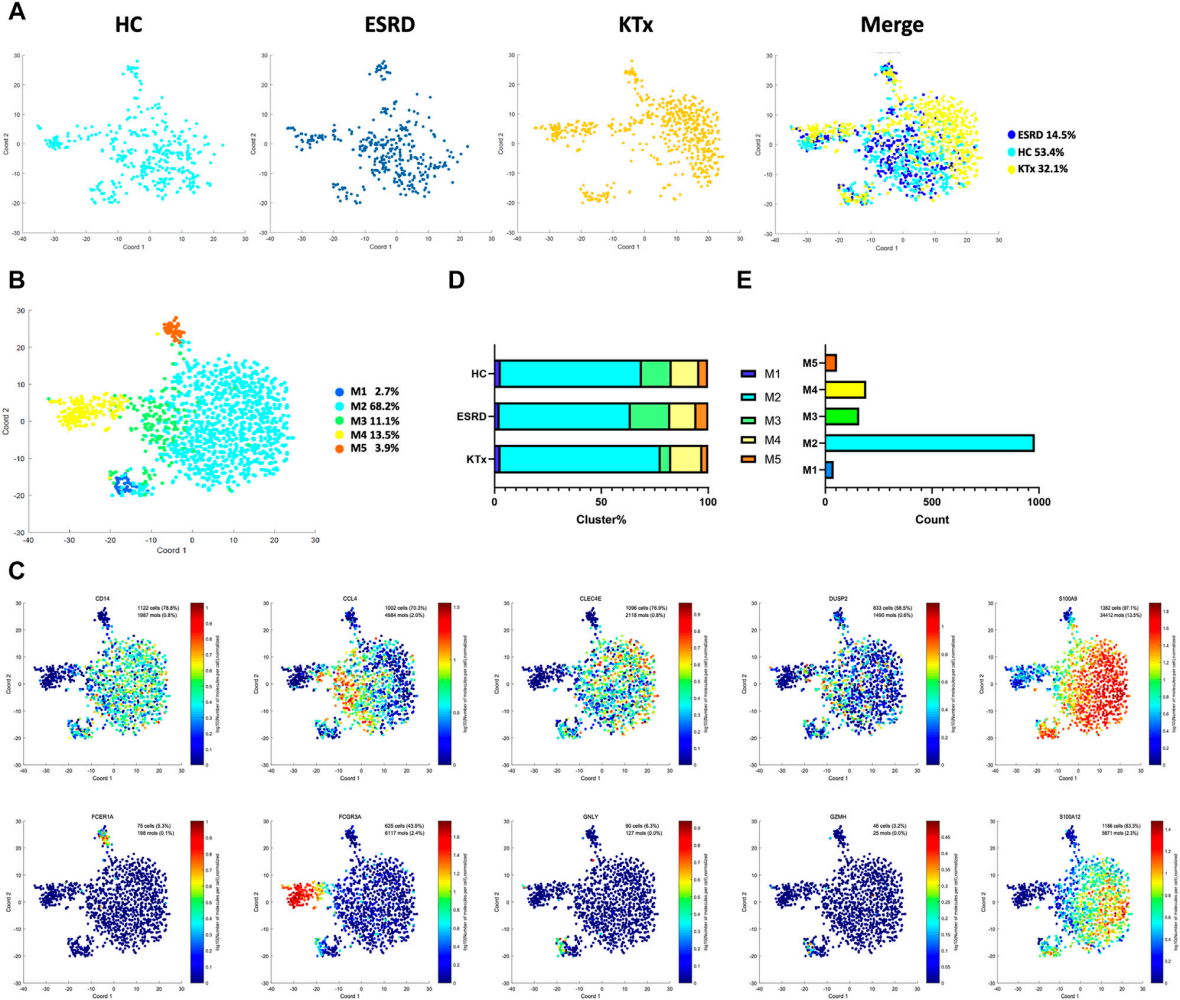
Myeloid clusters in PBMCs from HC, ESRD, and KTx. **(A,B)** tSNE of single-cell profile with each cell color coded for myeloid subsets. **(C)** Expression of highly expressed genes in each myeloid cluster. **(D)** Proportion of 5 myeloid subsets. **(E)** Cell count of 5 myeloid subsets.

Five myeloid cell subpopulations were defined by scRNA-seq (Figures 4A,B) given as follows: M1 cluster (GZMH^+^GNLY^+^), M2 cluster (S100A12^+^S100A9^+^), M3 cluster (CCL4^+^DUSP2^+^), M4 cluster (FCGR3A^+^), and M5 cluster (FCER1A^+^CD1c^+^) (Figure 4D). The gene expression pattern of the M5 cluster was relatively close to that of dendritic cells (DCs) (García-González et al., 2017). The specific marker distribution and the distribution in each cluster are shown in Figure 4C and Supplementary Figures 5A,B. There was a significant difference in the M3 cluster between the KTx and HC groups (Figure 4E), and the results of KEGG and GO enrichment analyses of highly expressed genes in the M3 subset showed that this cluster was related to cytokine-mediated signaling pathways (Supplementary Figure S5C). Unfortunately, we did not verify this difference by flow cytometry (Supplementary Figure S5D).

### IGLC3^low^IGKC^high^TCL1A^-^ B Cells Were Significantly Suppressed in KTx Patients

**FIGURE 5:**
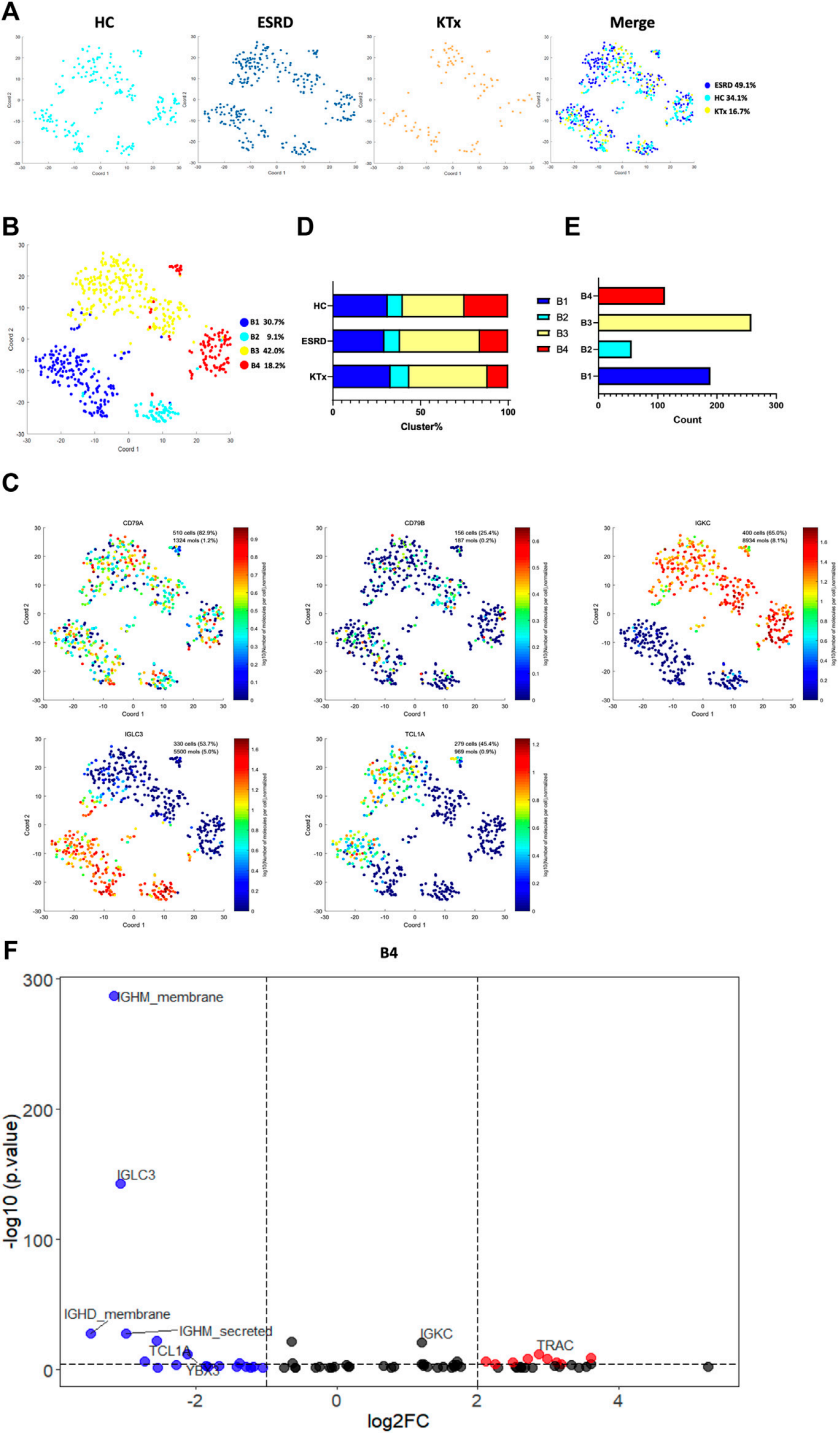
B-cell clusters in PBMCs from HC, ESRD, and KTx. **(A,B)** tSNE of single-cell profile with each cell color coded for B-cell subsets. **(C)** Expression of highly expressed genes in each B-cell cluster. **(D)** Distribution of 5 B-cell subsets. **(E)** Cell count of 4 B-cell subsets. **(F)** Gene fingerprint expression volcano map of B4 cluster.

**FIGURE 6:**
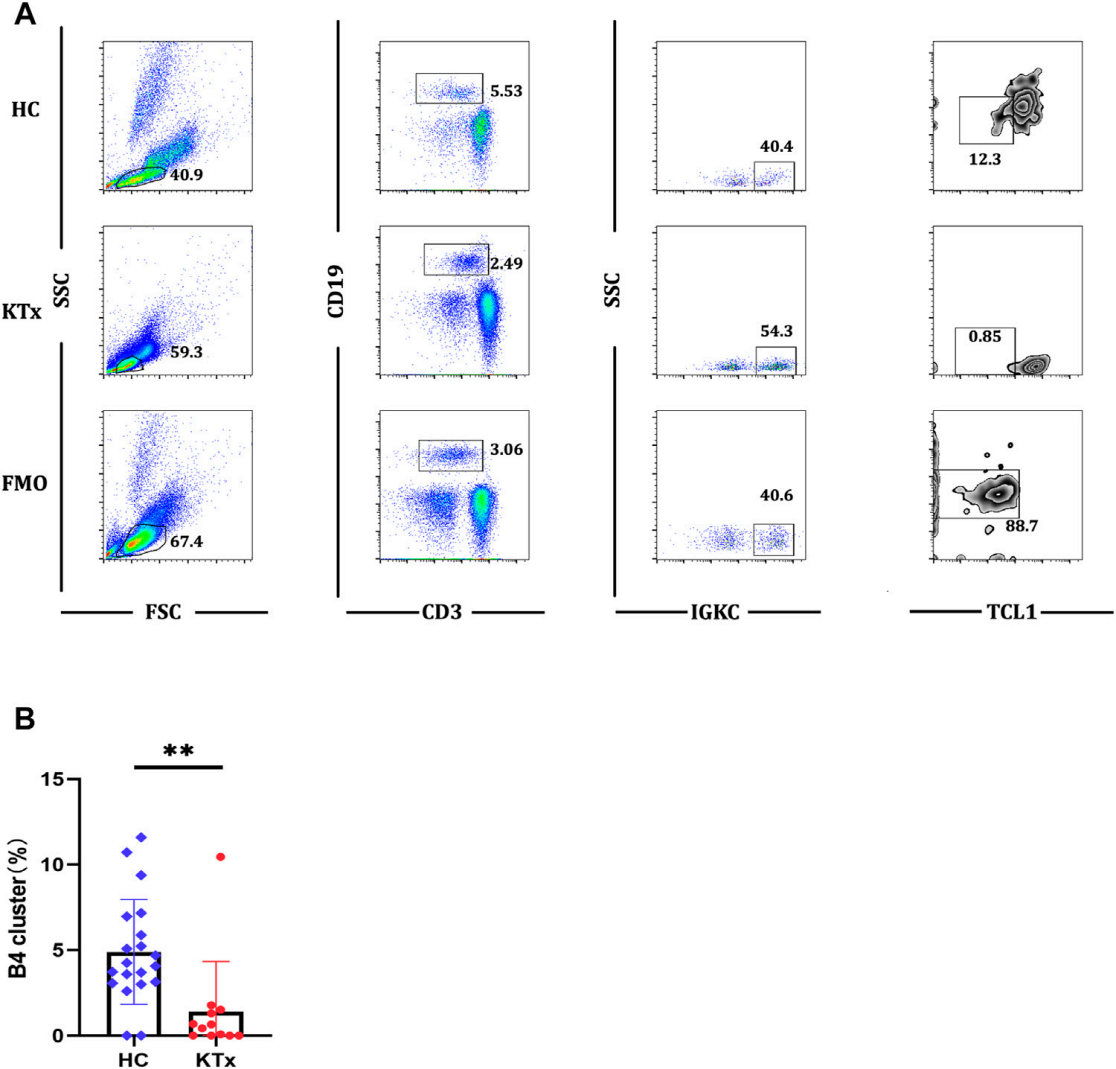
Flow cytometric analysis of B4 cluster in HC and KTx groups. **(A)** Gating strategy and flow cytometric plots of B4 cluster in HC, KTx, and FMO control groups. **(B)** Frequency of CD19^+^IGKC^+^IGLC^-^B4 cluster from CD19 ^+^ B cells in HC and KTx groups. A Mann–Whitney test was used to analyze the differences between two groups. **p < 0.01.

Four B-cell subpopulations were defined by scRNA-seq (Figures 5A,B) given as follows: the B1 cluster (IGLC3^high^IGKC^low^TCL1A^+^), B2 cluster (IGLC3^high^IGKC^low^TCL1A^−^), B3 cluster (IGLC3^low^IGKC^high^TCL1A^+^), and B4 cluster (IGLC3^low^IGKC^high^TCL1A^−^). The specific markers and distribution in each cluster are shown in Figure 5C,D, Supplementary Figures S6A,B. Interestingly, our analysis showed that the KTx group had a significantly lower B4 percentage than the HC group (Figure 5E). To further verify this change, we detected the proportion of B4 cells by flow cytometry and found that the B4 cluster (IGLC3^low^IGKC^high^TCL1A^-^) was significantly reduced in the KTx group compared with the HC group (Figures 6A,B). In addition, the B4 cluster highly expressed the characteristic T-cell gene TRAC (Figure 5F).

### IGLC3^low^IGKC^high^TCL1A^-^ B Cells Might Have Potential Activated and Proliferous Effects

**FIGURE 7:**
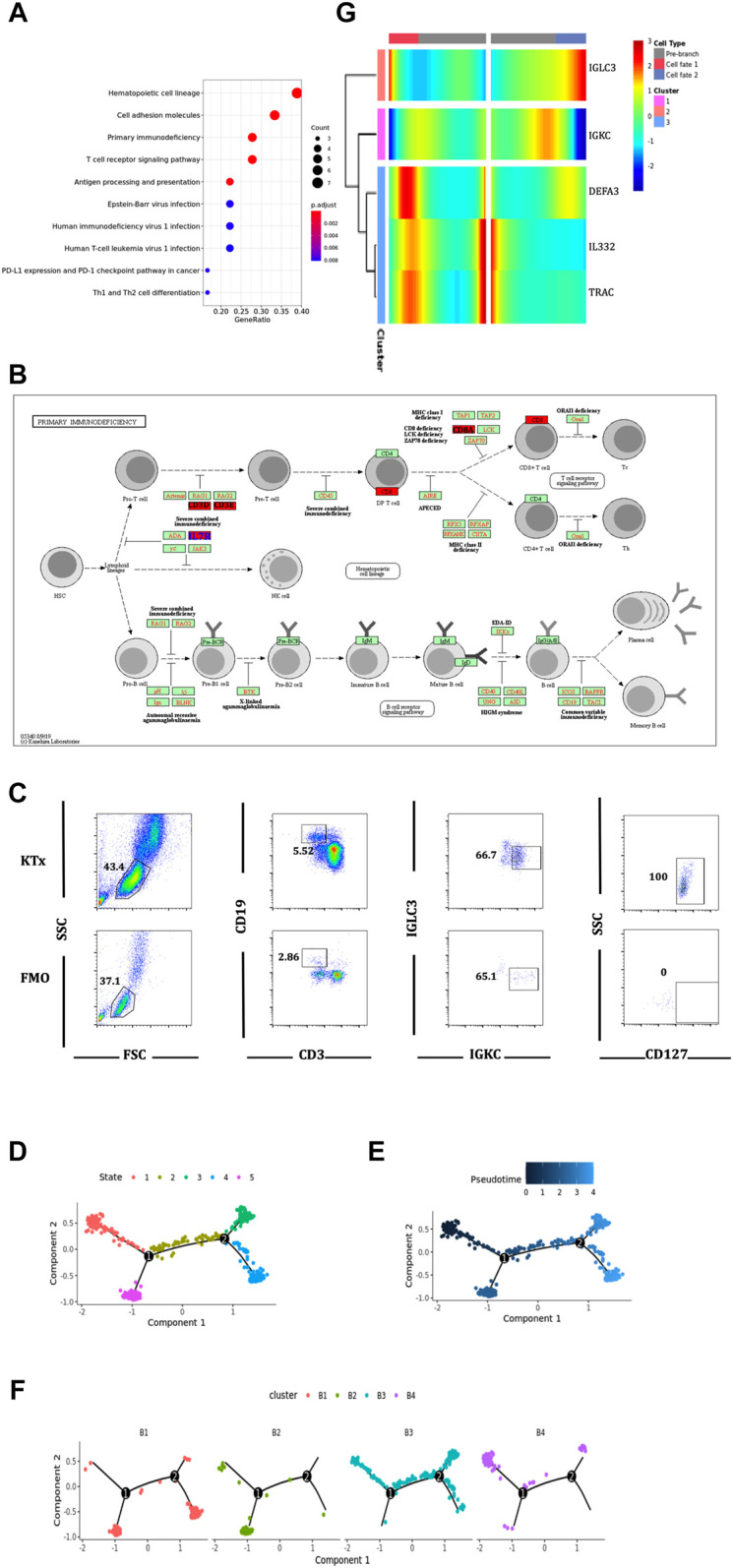
Function and differentiation analysis of B4 cluster. **(A)** KEGG analysis of highly expressed genes on B4 cluster. **(B)** In the primary immunodeficiency pathway, IL7R was the key gene. **(C)** Flow cytometric analysis of CD19^+^IGKC^+^IGLC^-^CD127^+^ B cells from lymphocytes in KTx group. **(D–F)** Packages monocle2 was used for pseudotime analysis of B-cell subset differentiation. **(G)** BEAM analysis of B4 cluster fate point.

To further verify the function of the B4 cluster (IGLC3^low^IGKC^high^TCL1A^-^), we performed the KEGG pathway analysis and found that the B4 cluster was closely related to primary immunodeficiency (Figure 7A). Further investigation found that IL7R, also known as CD127, was the key gene (Figure 7B). CD127 plays an important role in the development of T cells and B cells, and positively regulate the survival and the response to antigens of T cells (Dooms, 2013). We found that the expression of CD127 on B4 cluster in the KTx group was very obvious compared with that observed for the fluorescence minus one (FMO) control (Figure 7C). The high expression of CD127 showed that B4 cluster represented as the activated and proliferous B-cell subset, which is inhibited in kidney recipients caused by immunosuppressant treatment.

The pseudotime analysis was used to analyze the “physical relationship” among the B-cell subsets. B cells could be divided into five fate states (Figures 7D,E). Among them, the B4 cluster (IGLC3^low^IGKC^high^TCL1A^−^) was regarded as an end point of the genetic relationship (Figure 7F) and was directly differentiated from the B3 cluster (IGLC3^low^IGKC^high^TCL1A^+^) (Figure 7F). The BEAM analysis was used to explore the key compartments necessary for B4 cluster differentiation. At the fate decision point 2, IL32 and TRAC were specifically marked as hot genes (Figure 7G).

## Discussion

Using PBMC scRNA-seq, flow cytometric, and bioinformatic analyses, we found that in renal allograft patients with immune accommodation, T, myeloid, and B cells but not NK cells could be affected by immunosuppressants. Among these cell types, a newly identified B-cell subset, the B4 cluster (CD19^+^IGLC3^low^IGKC^high^TCL1A^-^CD127^+^), was affected the most. More importantly, the expression of IL7R (CD127) in this subgroup showed that the B4 cluster might have an activated potential.

With its high resolution, single-cell sequencing greatly improves the ability to identify rare immune cells and can more comprehensively describe the functions of immune cells (Nguyen et al., 2018). At present, studies have shown the important roles of B-cell subpopulations in inflammatory bowel disease and noted that B cells significantly remodel the phenotype in the course of disease development (Castro-Dopico et al., 2020). In addition, Park et al. (2021) found that in *Dabie bandavirus*–infected individuals, B cells are greatly expanded and that SFTSV infection can suppress the maturation of high-affinity antibodies and inhibit neutralizing antibodies secreted by plasma B cells, leading to a large amount of viral replication and subsequent death. In addition, through the single-cell sequencing technology, Sutton et al. (2021) found that SARS-CoV exists in malaria-infected patients. The phenotypic B-cell subpopulation was characterized by a CD21^−^CD27^−^ or CD21^−^CD27^+^ phenotype, which represents an immune-exhausted phenotype. These findings represent the contribution of single-cell sequencing to the understanding of the functional diversity of B cells, especially in immune-related diseases.

Kidney transplantation is the most common solid organ transplantation operation. Due to the implantation of an allograft and the use of immunosuppressive agents, kidney transplantation has a relatively great impact on the recipient’s immune system. In fact, some researchers have already used single-cell sequencing to test the immune status of kidney transplant recipients with rejection. Varma et al. (2021) found that in rejected renal allografts, allogeneic infiltrated myeloid cells differentiated from monocytes into proinflammatory macrophages. The trajectory analysis showed a unique interaction with allogeneic renal parenchymal cells, and the Axl expression on myeloid cells played a major role in promoting intragraft myeloid cell and T-cell differentiation. Lei et al. (2020) identified the most important pathogenic effect of cathepsin S (Cat-S)–expressing monocytes by single-cell sequencing. In addition, Liu et al. (2020) found increased collagen- and extracellular matrix component-expressing myofibroblasts in the human kidneys undergoing chronic rejection, which indicates that renal fibrosis plays an important pathological role in rejection. These findings not only deepen our understanding of the immunoregulatory process in allograft rejection but also indicate the great potential of single-cell sequencing technology in investigating rare and novel immune cell subgroups and analyzing the interactions between immune cells and transplant parenchymal cells.

In our study, we focused on the so-called immune accommodation state in kidney transplant recipients to obtain a deeper understanding of the immune status of these recipients. Here, we discovered a new type of B-cell subgroup (IGLC3^low^IGKC^high^TCL1A^−^). This subpopulation is characterized by a unique IG chain composition and a low expression of TCL1A, which is considered a stable B-cell marker in kidney transplant patients (Heidt et al., 2015). In addition, IGKC is regarded as a prognostic marker in breast cancer (Pandey et al., 2014), which means that the genotype of the light chain region may be related to the immune status and function of B cells.

In the IGLC3^low^IGKC^high^TCL1A^−^ B-cell cluster, a small number of cells were found to express IL7R (CD127). IL7 provides survival signals to T cells and plays an important role in autoimmune diseases. Also, IL7R is indispensable for B-cell lymphopoiesis. Alhaj Hussen et al. (2017) reported the existence of CD127^+^ early lymphoid progenitors can differentiate into NK cells, ILCs, and B cells but lack the potential to differentiate into T cells. IL7 and IL7Rα–deficient mice showed arrested B-cell development in the bone marrow. An early lymphocyte expansion is severely impaired in IL7R–deficient mice, and lymphopenia in IL7 gene-knockout mice identified IL7 as a nonredundant cytokine. This proved that CD127^+^ B cells may represent a B-cell subgroup with a proliferation potential and suggest that the suppressive effect of B4 subset may be the reason for the decrease in the proportion and absolute count of B cells in kidney transplant recipients. Furthermore, we explored whether there are interventions that can induce the differentiation of IGLC3^low^IGKC^high^TCL1A^−^ B cells. As mentioned above, through pseudotime and BEAM analysis, we discovered the important roles of IL32 and TRAC in this process. In particular, researchers have reported that IL32γ enhances host immunity to *Mycobacterium tuberculosis* in mice. Besides, increased IL32 was found in giant cell arteritis with enhanced B-cell survival and expansion. Although there is currently no complete evidence to prove the causality between IL32 and B cells, but we predicted that IL32 might be a potential proliferation factor for B cells. Correspondingly, we did not find any reports on TRAC in B cells, so the functions and roles of TRAC in the differentiation and development of B cells need to be further explored.

There were also some limitations to our study. We did not conduct further *in vitro* experiments to verify the activated potential of CD19^+^IGLC3^low^IGKC^high^TCL1A^-^CD127^+^ B cells and evaluate whether IL32 could induce CD19^+^IGLC3^low^IGKC^high^TCL1A^-^CD127^+^ B-cell differentiation. We will try to investigate these issues in our future research. In addition, there was only one specimen included in each single-cell sequencing group, but we did verify most of our findings through multiple-sample flow cytometry.

## Conclusion

By scRNA-seq, flow cytometric, and bioinformatic analyses, we found that the level of a novel B-cell subset (CD19^+^IGLC3^low^IGKC^high^TCL1A^−^CD127^+^) in renal transplant recipients with accommodation was significantly lower than that in healthy people and that this B-cell subset showed an activated potential because of its high expression of CD127. This study might reveal the effects of IS commonly used in clinical practice on B-cell subsets and the related mechanism.

## Data Availability

The data presented in the study are deposited in the NCBI Gene Expression Omnibus database repository (https://www.ncbi.nlm.nih.gov/geo/query/acc.cgi?accGSE175429), accession number (GSE175429).

## References

[B1] Alhaj Hussen K. Vu Manh T. P. Guimiot F. Nelson E. Chabaane E. Delord M. (2017). Molecular and Functional Characterization of Lymphoid Progenitor Subsets Reveals a Bipartite Architecture of Human Lymphopoiesis. Immunity. 47 (4), 680–696.e8. 10.1016/j.immuni.2017.09.009 29045900

[B2] Castro-Dopico T. Colombel J. F. Mehandru S. (2020). Targeting B Cells for Inflammatory Bowel Disease Treatment: Back to the Future. Curr. Opin. Pharmacol. 55, 90–98. 10.1016/j.coph.2020.10.002 33166872 PMC7894973

[B3] Dooms H. (2013). Interleukin-7: Fuel for the Autoimmune Attack. J. Autoimmun. 45, 40–48. 10.1016/j.jaut.2013.06.007 23831438

[B4] Dulken B. W. Buckley M. T. Navarro Negredo P. Saligrama N. Cayrol R. Leeman D. S. (2019). Single-cell Analysis Reveals T Cell Infiltration in Old Neurogenic Niches. Nature. 571 (7764), 205–210. 10.1038/s41586-019-1362-5 31270459 PMC7111535

[B5] Fishman J. A. (2013). Opportunistic Infections-Ccoming to the Limits of Immunosuppression? Cold Spring Harb Perspect. Med. 3 (10), a015669. 10.1101/cshperspect.a015669 24086067 PMC3784816

[B6] Garcia de Mattos Barbosa M. Cascalho M. Platt J. L. (2018). Accommodation in ABO-Incompatible Organ Transplants. Xenotransplantation 25 (3), e12418. 10.1111/xen.12418 29913044 PMC6047762

[B7] García-González P. A. Schinnerling K. Sepúlveda-Gutiérrez A. Maggi J. Mehdi A. M. Nel H. J. (2017). Dexamethasone and Monophosphoryl Lipid A Induce a Distinctive Profile on Monocyte-Derived Dendritic Cells through Transcriptional Modulation of Genes Associated with Essential Processes of the Immune Response. Front. Immunol. 8, 1350. 10.3389/fimmu.2017.01350 29109727 PMC5660598

[B8] Heidt S. Vergunst M. Anholts J. D. Reinders M. E. de Fijter J. W. Eikmans M. (2015). B Cell Markers of Operational Tolerance Can Discriminate Acute Kidney Allograft Rejection from Stable Graft Function. Transplantation. 99 (5), 1058–1064. 10.1097/tp.0000000000000465 25340606

[B9] Lei Y. Ehle B. Kumar S. V. Müller S. Moll S. Malone A. F. (2020). Cathepsin S and Protease-Activated Receptor-2 Drive Alloimmunity and Immune Regulation in Kidney Allograft Rejection. Front Cel Dev Biol. 8, 398. 10.3389/fcell.2020.00398 PMC7290053 32582696

[B10] Lim M. A. Kohli J. Bloom R. D. (2017). Immunosuppression for Kidney Transplantation: Where Are We Now and where Are We Going? Transpl. Rev (Orlando). 31 (1), 10–17. 10.1016/j.trre.2016.10.006 28340885

[B11] Liu Y. Hu J. Liu D. Zhou S. Liao J. Liao G. (2020). Single-cell Analysis Reveals Immune Landscape in Kidneys of Patients with Chronic Transplant Rejection. Theranostics. 10 (19), 8851–8862. 10.7150/thno.48201 32754283 PMC7392010

[B12] Nguyen A. Khoo W. H. Moran I. Croucher P. I. Phan T. G. (2018). Single Cell RNA Sequencing of Rare Immune Cell Populations. Front. Immunol. 9, 1553. 10.3389/fimmu.2018.01553 30022984 PMC6039576

[B13] Pandey J. P. Kistner-Griffin E. Black L. Namboodiri A. M. Iwasaki M. Kasuga Y. (2014). IGKC and FcγR Genotypes and Humoral Immunity to HER2 in Breast Cancer. Immunobiology. 219 (2), 113–117. 10.1016/j.imbio.2013.08.005 24054945

[B14] Parajuli S. Mandelbrot D. A. Aziz F. Garg N. Muth B. Mohamed M. (2018). Characteristics and Outcomes of Kidney Transplant Recipients with a Functioning Graft for More Than 25 Years. Kidney Dis. (Basel). 4 (4), 255–261. 10.1159/000491575 30574502 PMC6276752

[B15] Park A. Park S. J. Jung K. L. Kim S. M. Kim E. H. Kim Y. I. (2021). Molecular Signatures of Inflammatory Profile and B-Cell Function in Patients with Severe Fever with Thrombocytopenia Syndrome. mBio. 12 (1), e02583. 10.1128/mBio.02583-20 33593977 PMC8545090

[B16] Pont F. Tosolini M. Fournié J. J. (2019). Single-Cell Signature Explorer for Comprehensive Visualization of Single Cell Signatures across scRNA-Seq Datasets. Nucleic Acids Res. 47 (21), e133. 10.1093/nar/gkz601 31294801 PMC6868346

[B17] Romano M. Fanelli G. Albany C. J. Giganti G. Lombardi G. (2019). Past, Present, and Future of Regulatory T Cell Therapy in Transplantation and Autoimmunity. Front. Immunol. 10, 43. 10.3389/fimmu.2019.00043 30804926 PMC6371029

[B18] Snijders M. L. H. Varol H. van der Zwan M. Becker J. U. Hesselink D. A. Baan C. C. (2020). Molecular Analysis of Renal Allograft Biopsies: Where Do We Stand and where Are We Going? Transplantation. 104 (12), 2478–2486. 10.1097/tp.0000000000003220 32150035

[B19] Sutton H. J. Aye R. Idris A. H. Vistein R. Nduati E. Kai O. (2021). Atypical B Cells Are Part of an Alternative Lineage of B Cells that Participates in Responses to Vaccination and Infection in Humans. Cell Rep. 34 (6), 108684. 10.1016/j.celrep.2020.108684 33567273 PMC7873835

[B20] Varma E. Luo X. Muthukumar T. (2021). Dissecting the Human Kidney Allograft Transcriptome: Single-Cell RNA Sequencing. Curr. Opin. Organ. Transpl. 26 (1), 43–51. 10.1097/mot.0000000000000840 33315769

[B21] Xiong X. Kuang H. Ansari S. Liu T. Gong J. Wang S. (2019). Landscape of Intercellular Crosstalk in Healthy and NASH Liver Revealed by Single-Cell Secretome Gene Analysis. Mol. Cel. 75 (3), 644–660.e5. 10.1016/j.molcel.2019.07.028 PMC7262680 31398325

[B22] Yu G. Wang L. G. Han Y. He Q. Y. (2012). clusterProfiler: an R Package for Comparing Biological Themes Among Gene Clusters. OMICS. 16 (5), 284–287. 10.1089/omi.2011.0118 22455463 PMC3339379

[B23] Zhuang Q. Li H. Yu M. Peng B. Liu S. Luo M. (2020). Profiles of B-Cell Subsets in Immunologically Stable Renal Allograft Recipients and End-Stage Renal Disease Patients. Transpl. Immunol. 58, 101249. 10.1016/j.trim.2019.101249 31626945

[B24] Zhuang Q. Peng B. Wei W. Gong H. Yu M. Yang M. (2019). The Detailed Distribution of T Cell Subpopulations in Immune-Stable Renal Allograft Recipients: a Single center Study. PeerJ. 7, e6417. 10.7717/peerj.6417 30775184 PMC6369828

