# Supplementary material for: Single-Cell Transcriptomic Analysis of Peripheral Blood Reveals a Novel B-Cell Subset in Renal Allograft Recipients With Accommodation
Source: Front Pharmacol. 2021 Sep 30;12:706580. doi: 10.3389/fphar.2021.706580 (PMC8514638; doi:10.3389/fphar.2021.706580)
Supplement: Supplementary file 1 [file Table1.DOCX]

| **Supplementary table 1. Baseline data of patients for flow cytometry** | | | |
| --- | --- | --- | --- |
|  | Healthy control | Kidney recipients | P |
| Age(years) | 29.95±3.79 | 43.83±6.96 | < 0.0001 |
| Gender(M/F) | 19/1 | 7/5 | 0.0185 |
| Time after operation (day) | / | 390.33±56.26 | / |
| Immunosuppressant | / | Tacrolimus+MMF+Prednisone | / |
| WBC（10^9/L） | / | 8.80±2.29 | / |
| Cr（μmol/L） | / | 102.33±21.68 | / |
| FK506(ng/mL) | / | 7.89±1.89 | / |
