# Supplementary material for: Single-Cell Transcriptomic Analysis of Peripheral Blood Reveals a Novel B-Cell Subset in Renal Allograft Recipients With Accommodation
Source: Front Pharmacol. 2021 Sep 30;12:706580. doi: 10.3389/fphar.2021.706580 (PMC8514638; doi:10.3389/fphar.2021.706580)
Supplement: Supplementary file 2 [file Table2.DOCX]

| **Supplementary table 2. Information of patients for Sc-RNA seq** | | | |
| --- | --- | --- | --- |
|  | Healthy control | Kidney recipients | ESRD |
| Age(years) | 30 | 32 | 35 |
| Gender | Male | Male | Male |
| Time after operation(day) | / | 393 | / |
| Immunosuppressant | / | Tacrolimus+MMF+Prednisone | / |
