# Supplementary material for: Single-Cell Transcriptomic Analysis of Peripheral Blood Reveals a Novel B-Cell Subset in Renal Allograft Recipients With Accommodation
Source: Front Pharmacol. 2021 Sep 30;12:706580. doi: 10.3389/fphar.2021.706580 (PMC8514638; doi:10.3389/fphar.2021.706580)
Supplement: Supplementary file 3 [file DataSheet1.DOCX]

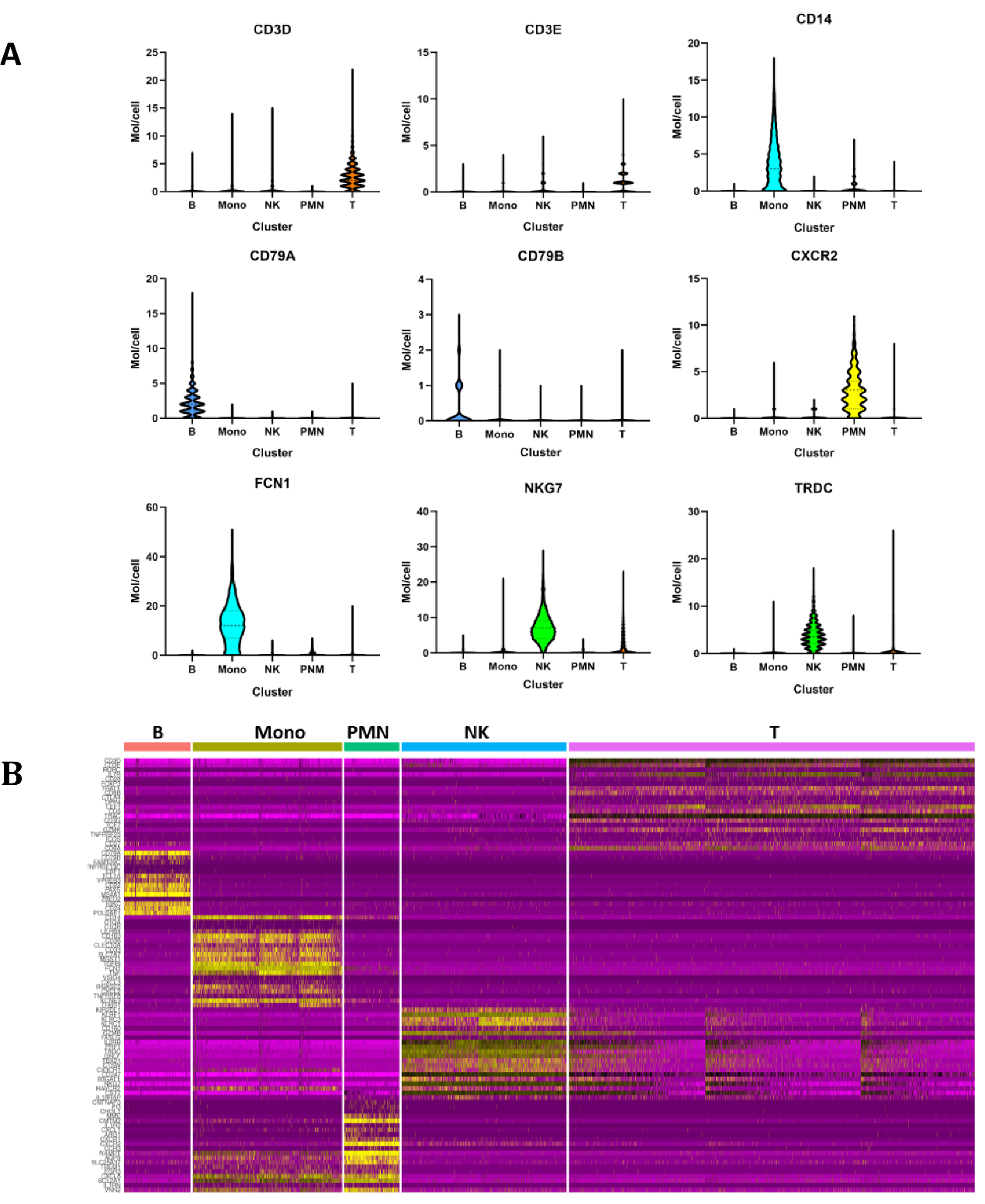


**Supplementary Figure 1. A:** The expression of the traditional marker used for PBMCs defined above. **B:** Differential expression analysis (heatmap) was performed comparing cells from 3 donors in each cluster.

**
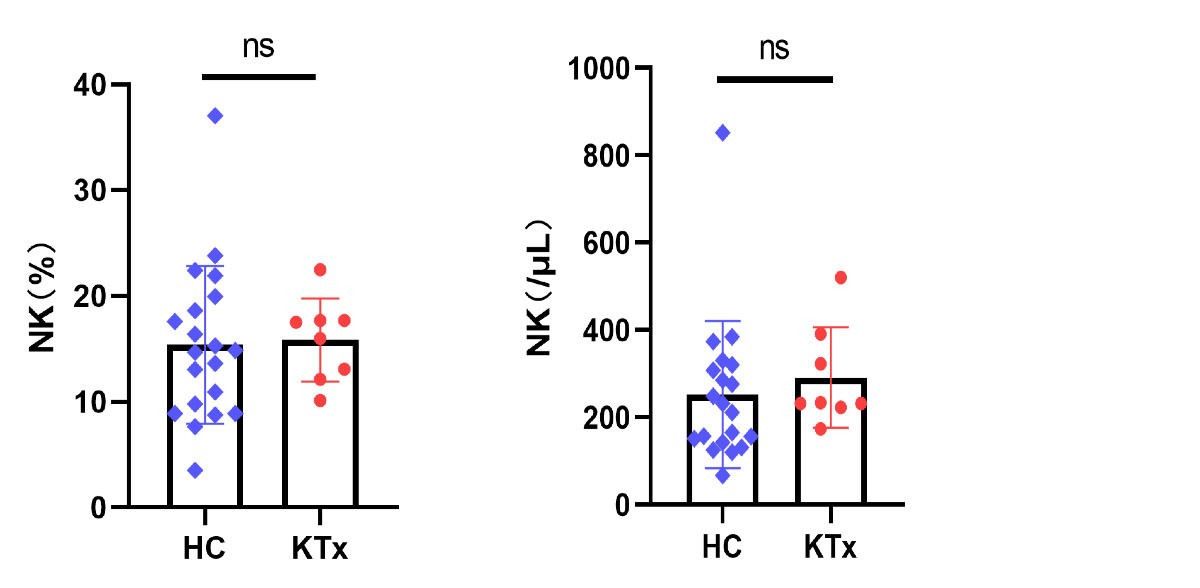
**

**Supplementary Figure 2.** The ratio and absolute number of NK cells in HC and KTx groups.


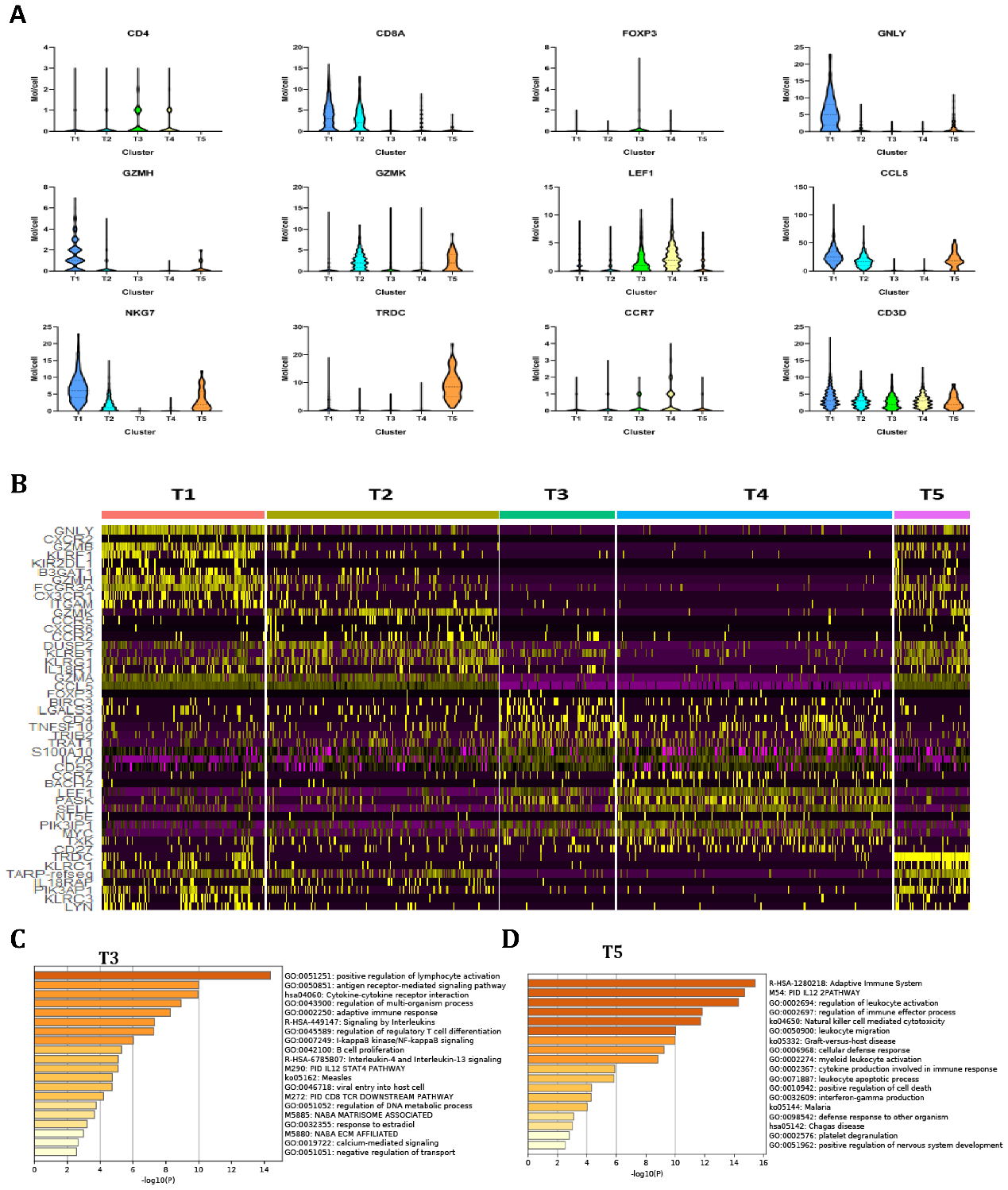


**Supplementary Figure 3. A:** The expression of highly expressed genes in each T-cell cluster. **B:** Heatmap of gene expressions in 5 T-cell clusters. **C:** The result of KEGG and GO analyze of high expression genes in T3. **D:** The result of KEGG and GO analyze of high expression genes in T5.


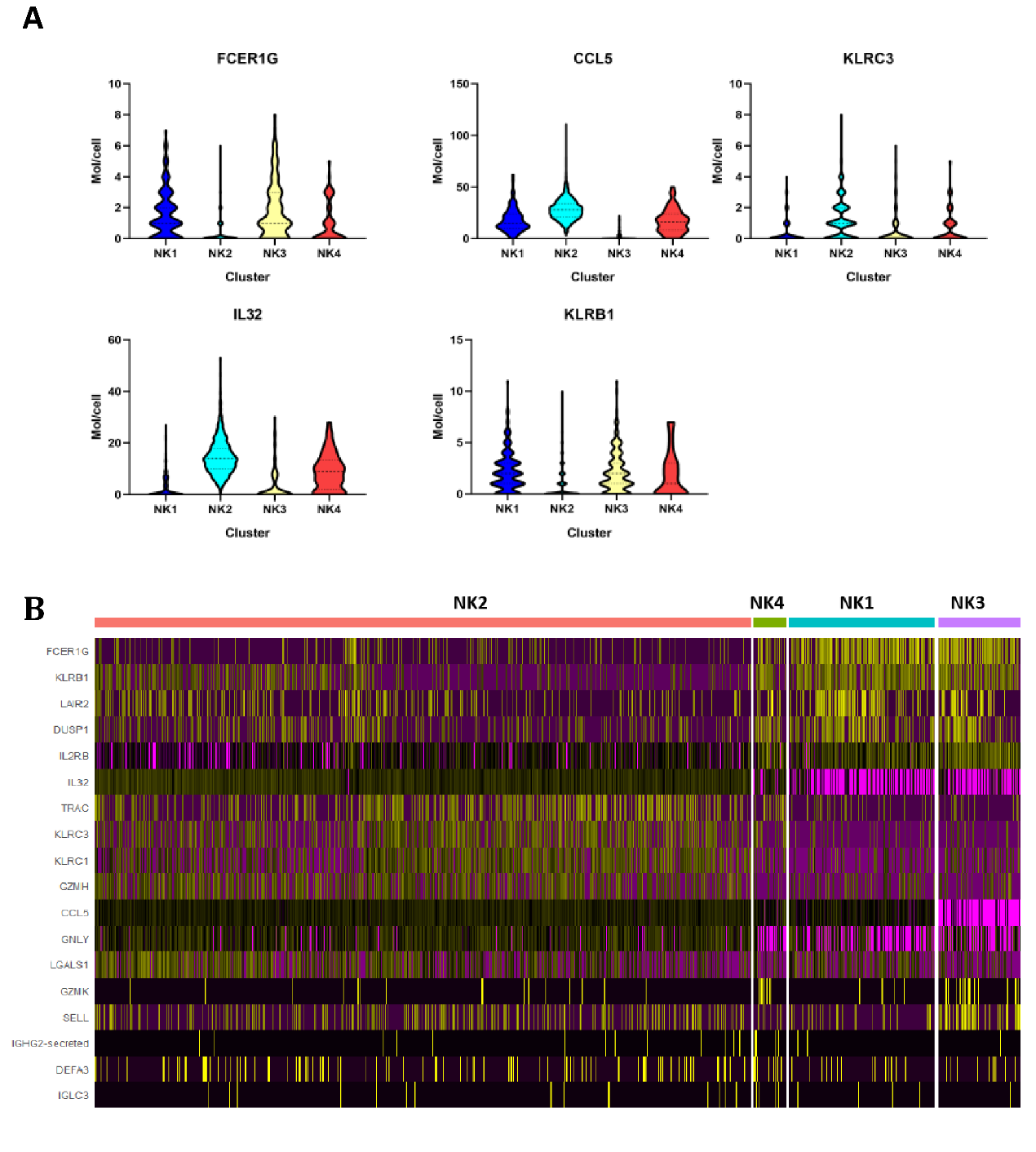


**Supplementary Figure 4. A:** The expression of highly expressed genes in each NK-cell cluster. **B:** Heatmap of gene expressions in 4 NK-cell clusters.


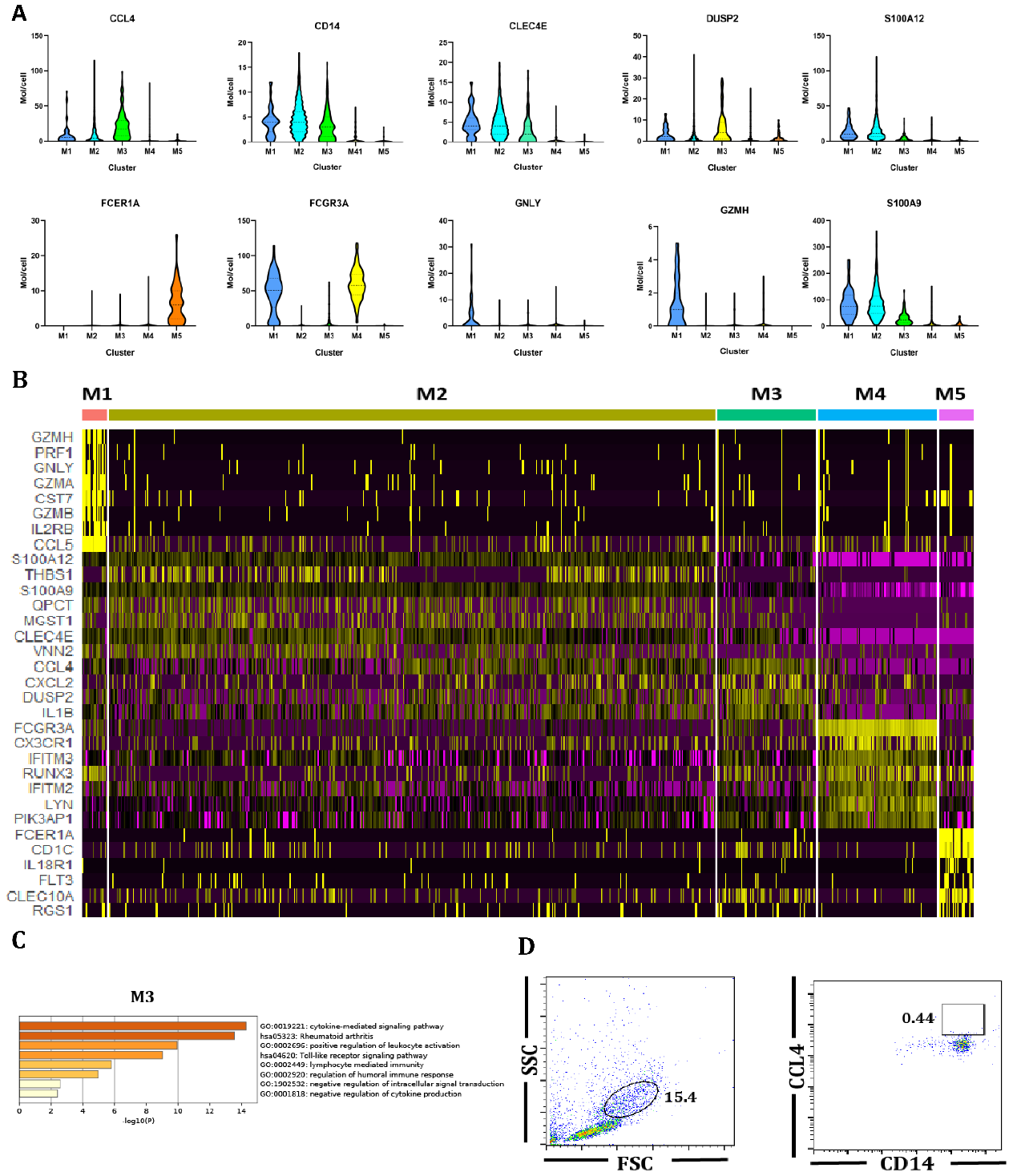


**Supplementary Figure 5. A:** The expression of highly expressed genes in each myeloid-cell cluster. **B:** Heatmap of gene expressions in 5 myeloid-cell cluster. **C:** The result of KEGG and GO analyze of high expression genes in M3. **D:** Gating strategy and flow cytometric plots of CD14+CCL4+ M3 monocyte cluster.


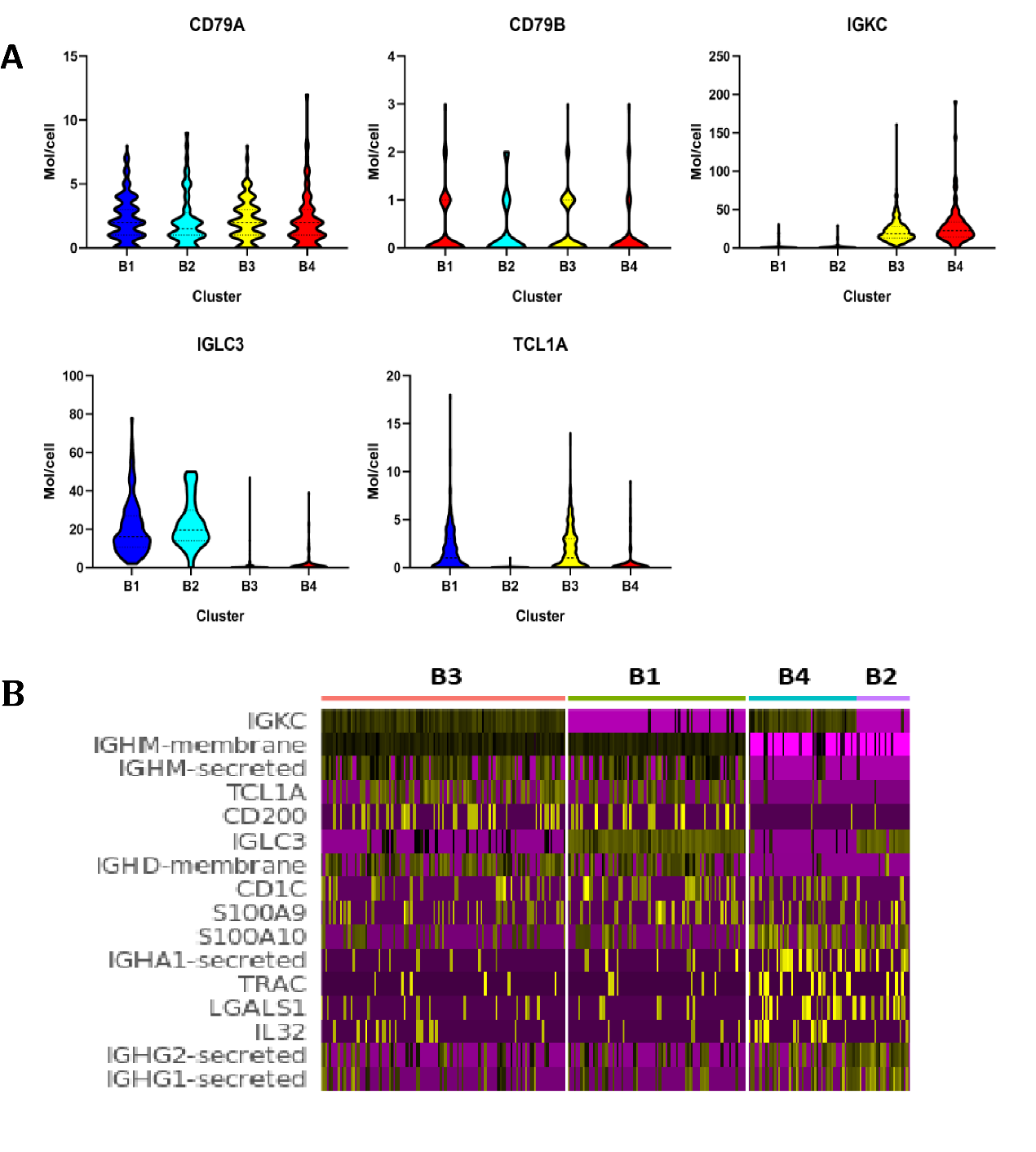


**Supplementary Figure 6. A:** The expression of highly expressed genes in each B-cell cluster. **B:** Heatmap of gene expressions in 5 B-cell cluster.
